# Soil Acidification Aggravates the Occurrence of Bacterial Wilt in South China

**DOI:** 10.3389/fmicb.2017.00703

**Published:** 2017-04-25

**Authors:** Shili Li, Yongqin Liu, Jiao Wang, Liang Yang, Shuting Zhang, Chen Xu, Wei Ding

**Affiliations:** ^1^Laboratory of Natural Products Pesticides, Southwest UniversityChongqing, China; ^2^Chongqing Tobacco Science Research InstituteChongqing, China

**Keywords:** soil acidification, bacterial wilt, *Ralstonia solancearum*, antagonistic bacteria, lime, wood ash

## Abstract

Soil acidification is a major problem in modern agricultural systems and is an important factor affecting the soil microbial community and soil health. However, little is known about the effect of soil acidification on soil-borne plant diseases. We performed a 4-year investigation in South China to evaluate the correlation between soil acidification and the occurrence of bacterial wilt. The results showed that the average soil pH in fields infected by bacterial wilt disease was much lower than that in non-disease fields. Moreover, the proportion of infected soils with pH lower than 5.5 was much higher than that of non-infected soils, and this phenomenon became more obvious as the area of bacterial wilt disease increased at soil pH lower than 5.5 from 2011 to 2014. Then, in a field pot experiment, bacterial wilt disease developed more quickly and severely in acidic conditions of pH 4.5, 5.0, and 5.5. These results indicate that soil acidification can cause the outbreak of bacterial wilt disease. Further experiments showed that acidic conditions (pH 4.5–5.5) favored the growth of the pathogen *Ralstonia solanacearum* but suppressed the growth and antagonistic activity of antagonistic bacteria of *Pseudomonas fluorescens* and *Bacillus cereus*. Moreover, acidic conditions of pH 5.5 were conducive to the expression of the virulence genes *PopA, PrhA*, and *SolR* but restrained resistance gene expression in tobacco. Finally, application of wood ash and lime as soil pH amendments improved soil pH and reduced the occurrence of bacterial wilt. Together, these findings improve our understanding of the correlation between soil acidification and soil-borne plant diseases and also suggest that regulation of soil acidification is the precondition and foundation of controlling bacterial wilt.

## Introduction

Rapid soil acidification has become a serious global problem and limits the sustainable development of modern agriculture. Forty to fifty percentage of the potentially arable lands were acidic, and about 60% of acid soils covered at least 48 developing countries distributed mainly in the tropics and subtropics (Narro et al., 2001; Kochian et al., 2015). Significant soil acidification was also observed in major croplands, grasslands, and forests in recent decades in China (Wang et al., 2007; Zhao Y. et al., 2009; Guo et al., 2010). Lands affect by acidity was estimated at 200 million ha, representing ~23% of total land area of China (Wang et al., 2015). An important cause of the significant pH decline in cropland is the irrational use of nitrogenous (N) fertilizer, the acidification driven by which is at least 10–100 times greater than that caused by acid rain (Guo et al., 2010).

Soil pH is one of the most influential factors in soil and has been called the master variable of soil. Thus, changes in soil pH can strongly affect many chemical, physical, and biological properties and processes in soil, especially the activity and structure of soil microbes. On one hand, soil pH is the best predictor of changes in soil bacterial communities, and bacterial relative abundance and diversity is positively and strongly affected by soil pH (Hartman et al., 2008; Lauber et al., 2009; Rousk et al., 2010a; Zhalnina et al., 2015), the latter nearly doubling between pH 4 and 8 (Rousk et al., 2010a). This positive correlation does not only affect the overall bacterial community composition but supports individual bacterial group compositions (Lauber et al., 2009). In contrast, soil pH has no effect on the relative abundance of fungi and has only a slight influence on fungal diversity (Rousk et al., 2010a). In lower pH conditions, the growth of bacteria is suppressed while the growth of fungi is increased (Rousk et al., 2009, 2010b), and when the soil pH is less than 4.5, both bacterial and the fungal growth are inhibited due to release of free aluminum (Al) or a decrease in plant productivity (Rousk et al., 2009). Another study showed that the fungal/bacterial ratio decreases significantly with increasing soil pH, but the fungal/bacterial biomass index increases slightly with increasing soil pH (Bååth and Anderson, 2003). However, soil micro-ecology balance and microbial diversity are necessary to maintain a healthy soil and to suppress plant diseases (Garbeva et al., 2004; Raaijmakers et al., 2009). Therefore, decreased pH or soil acidification has a direct influence on microbial diversity and the soil ecosystem, leading to an imbalance in the soil micro-ecosystem and an abundance of soil-borne diseases in arable soil.

On the other hand, the ambient pH levels determine the capability of the pathogen to colonize, invade, and kill the host successfully (Alkan et al., 2013). They also regulate the synthesis of pathogenesis factors and affect the expression of virulence and survival related genes (Manteau et al., 2003). Among the soil borne pathogens, the virulence of *Gaeu-mannomyces graminis* var. *Tritici (Ggt)* is more closely related to environment pH. And severity of take-all on wheat was significantly lower at low pH (5.0) than at higher pH (Ownley et al., 1992). Similarly, the expression of *Ggt* pathogenesis-related genes depends on the ambient pH. In three *Ggt* strains, the *exo* mRNA levels were up-regulated in acidic conditions (pH 4.6) than in neutral conditions (Daval et al., 2013). Furthermore, the expression of *AaK1* gene in *Alternaria alternata* exhibited significantly lowered virulence at pH 6.0 (Eshel et al., 2002). In addition, pH also has a profound influence on nutrient uptake, ion toxicity, plant growth, and function. In general, environment factors could affect the pathogenicity of microorganism and the resistance of plant simultaneously during pathogenic microorganism-host plant interactions. Therefore, determination of changes in pathogenicity of *Ralstonia solanacearum* and resistance of tobacco plants under different pH condition would help to explain why soil borne disease occurs frequently in the area of soil acidification.

Bacterial wilt, which is caused by the invasive microorganism *R. solanacearum*, is a typical soil-borne plant disease with a wide geographical distribution (Genin, 2010). *R. solanacearum* infects plant roots through wounds or natural fissures, colonizes the plant vasculature, and produces a large amount of extracellular polysaccharides (EPS), leading to wilting and death of the host plant (Schell, 2000). After the host plant is destroyed, the bacterium returns to the soil through saprophytism until it contacts new hosts (Genin, 2010). As a type of soil-borne plant disease, the occurrence and epidemics of bacterial wilt are tightly correlated with the physical and chemical properties of soil. Based on 10 years of continuous tracking, we found that the occurrence of bacterial wilt was becoming increasingly serious in solanaceous croplands in mountainous southwest China. Significant soil acidification was also observed in major Chinese croplands in almost the same period. While previous studies have demonstrated that a decrease in soil pH could weaken plant growth and change the microbial community and activity, little is known about how a soil-borne plant disease responds to soil acidification. In other words, we cannot determine whether soil pH itself is an important factor in the occurrence of the soil-borne plant disease bacterial wilt.

Our aims for this study were as follows: (i) to study whether the occurrence of bacterial wilt is closely related to significant soil acidification, (ii) to determine whether there is a positive relationship and to reveal the mechanism for why bacterial wilt is more serious in acidic soils, (iii) to test the effect of several pH amendments on the improvement of acidic soil and the reduction of bacterial wilt. We collected hundreds of soil samples, including both healthy and diseased soils, over 4 years to investigate the relationship between soil acidification and the serious occurrence of bacterial wilt. Then, we conducted a pot experiment to verify the relevance and to determine the direct impact of pH on the pathogen *R. solanacearum* and the representative antagonistic microorganisms *Pseudomonas fluorescens* and *Bacillus cereus* (Chakravarty and Kalita, 2012). Finally, we used two pH amendments, lime and wood ash, to improve the soil pH and evaluated their effect on the occurrence of bacterial wilt.

## Materials and methods

### Bacterial strains and growth conditions

An *R. solanacearum* wild-type strain CCT011 (phylotype I, race 1, biovar 3), isolated from wilting tobacco plants in Qianjiang, Chongqing, and two antagonistic bacteria, *P. fluorescens* and *B. cereus* (Duyi Biotechnology Co. Ltd., Shanghai), were used in this study. The *R. solanacearum* strain was grown at 30°C on NA plates (Li et al., 2016) or in B medium (10.0 g of bacto peptone, 1.0 g of yeast extract, 1.0 g of casamino acid, and 1,000 ml of distilled water). *P. fluorescens* and *B. cereus* were grown in Broth medium (3.0 g of beef extract, 5.0 g of NaCl, 10.0 g of peptone, and 1,000 ml of distilled water) at 30°C. When required, the pH was adjusted with 1 M of hydrochloric acid or 1 M of sodium hydroxide.

### Soils, sampling, and pH determination

A total of 652 soils were sampled from 11 provinces (Hunan, Guangdong, Jiangxi, Fujian, Sichuan, Yunnan, Guizhou, Anhui, Henan, Shandong, and Hubei) located mostly in southern China in 2011, 2012, 2013, and 2014. A total of 382 soil samples were collected from fields with severe bacterial wilt. The other 270 soil samples were collected from plots with no bacterial wilt. We sampled both infected soil and non-infected soil at one geographical sampling site, apart from a few exceptions. Most of the sampling plots were larger than 667 m^2^. Before sampling, we removed the humus layer using a plastic hand-rake and a scraper. Five to 10 small samples were taken from the major root zone (0–20 cm depth) of each individual plot using a shovel and were combined and stored in plastic bags in an icebox. After being transported to the laboratory, soil samples were sieved (<2 mm) and stored in a refrigerator at 4°C.

Soil pH was measured as previously described with some modifications (Pietri and Brookes, 2008). Briefly, 20 g of air-dry soil and 100 ml of deionized water, corresponding to a soil: water ratio of 1:5 (weight: volume), were mixed, shaken for 5 min, and left to settle for 30 min. Then, soil pH was measured using a pH meter (Sartorius PB-10, Beijing). Each soil sample was measured three times, and the average value was used for statistical analysis.

### Inoculation assay

To evaluate disease development under different pH conditions (4.5~8.0), we performed an indoor pot experiment. The pH of autoclaved seedling soil medium was adjusted using different concentrations of hydrochloric acid or sodium hydroxide. Three-week-old tobacco seedlings (Yunyan 97) were transferred into pots filled with pH-adjusted seedling soil medium, and they were watered with pH-adjusted water to create fixed pH conditions. After 3 weeks, individual plants were watered with 20 ml of 10^8^ CFU/ml bacterial suspension. Inoculated plants were incubated with a 12/12 h light/dark cycle at 28°C, and 15 plants were used for each pH treatment. Disease development was recorded daily on a disease index scale from 0 for no symptoms to 4 for completely dead plants.

We also performed a field pot experiment in Qianjiang, Chongqing, China. The soils used in this experiment were collected directly from the field, and the pH was adjusted as described above. Individual tobacco seedlings (Yunyan 97) were transplanted into large pots containing pH-adjusted soil. The soil pH was checked frequently using a soil pH meter and was adjusted with hydrochloric acid or sodium hydroxide as necessary. After 3 weeks, we inoculated the plants with 20 ml of 10^8^ CFU/ml bacterial suspension, and 15 plants were used for each pH treatment. Disease progress was scored every fifth day on a disease index scale from 0 to 9 (0, no wilt symptoms; 1, less than half of the leaves on disease side wilted; 3, half to two-thirds of the leaves on disease side wilted; 5, more than two-thirds of the leaves on disease side wilted; 7, all the leaves on disease side wilted; 9, the whole plant died). The DI was calculated using the following formula: DI = [Σ(*n*_i_ × *v*_i_)/(*N*×*V*)] × 100, where *n*_i_ = the number of plants with the respective disease index, *v*_i_ = disease index (0, 1, 3, 5, 7, or 9), *N* = the total number of plants used in each treatment, and *V* = the highest disease index (9).

### Growth adaptability of *R. solanacearum* and antagonistic bacteria to ambient pH

The growth curve of *R. solanacearum* under different pH conditions (4.0~8.5) was determined as follows. First, *R. solanacearum* cells were grown in B medium at 28°C for 12 h. The bacterial suspension (OD_600_ ≈ 1.0) was then transferred into pH-adjusted B medium at a 1:100 ratio. The OD_600_ of each treatment was measured using a spectrophotometer with a 4-h time interval. For the growth rates of *P. fluorescens* and *B. cereus*, 100 μl of bacterial suspension at 10^7^ CFU/ml was added to 50 ml of pH-adjusted Broth medium and shaken at 150 rpm for 48 h. The bacterial concentration was determined using a nephelometer. Three independent experiments were performed, and three replicates were used for each treatment in each independent experiment.

### Determination of the antagonistic activity of *B. cereus* against *R. solanacearum*

The antagonistic activity of *B. cereus* against *R. solanacearum* was evaluated on NA plates using the Oxford cup assay. pH-adjusted NA plates were evenly streaked with 100 μl of 10^8^ CFU/ml *R. solanacearum* suspension. A sterilized Oxford cup with a diameter of 10 mm was placed in the central of the plate, and 100 μl of 10^8^ CFU/ml *B. cereus* suspension was added to the Oxford cup. Inoculated plates were incubated at 30°C for 48 h. The inhibition zone around the Oxford cup was measured, and the average diameter of the inhibition zone was used as an indicator of antagonistic activity. Three replicates were used for each treatment (pH 4.0–8.5 at an interval of 0.5).

### Effect of different pH conditions on pathogen virulence and plant defense

To confirm the expression of virulence genes under different pH conditions, *R. solanacearum* was grown in B medium at varying pH (4.5~8.0), respectively. The cells were collected at the logarithmic phase (OD_600_ ≈ 0.8). Total RNA was extracted using the TRIZol regent method and was digested and purified using DNase I kits. Purified RNA (1 μg) was reverse-transcribed into cDNA using an iScript cDNA Synthesis kit (Bio-Rad, Hercules, CA, USA). Primers for the tested genes were designed based on the sequence of the *R. solanacearum* GM1000 genome. The primers sequences are listed in Table 1. *serC* was used as the housekeeping gene for data analysis (Li et al., 2016). Quantitative real-time analysis and gene expression data processing were performed as described by Li et al. (2016).

**Table 1:** **DNA primers used in this study**.

**Primers**	**Sequence (5′to 3′)**
*NtACC Oxidase_*F	GACAAAGGGACATTACAAGAAGT
*NtACC Oxidase_*R	GAGAAGGATTATGCCACCAG
*NtPR1a/c_*F	AACCTTTGACCTGGGACGAC
*NtPR1a/c_*R	GCACATCCAACACGAACCGA
*E3ligase_*F	TTCTCGGAGCCTCTTATG
*E3ligase_*R	CCCTCTTCCCACCTTGC
*Thaumatin_*F	TCACCCGTGGTATTAGG
*Thaumatin_*R	GTTCCTGTAGGACAAGCA
*UBI3_F*	GCCGACTACAACATCCAGAAGG
*UBI3_R*	TGCAACACAGCGAGCTTAACC

For resistant gene expression assays, tobacco was cultivated under hydroponic conditions in an artificial climate chamber at 26°C and 60% relative humidity for 7 weeks. Then, the plants were transferred into 250 ml flasks containing nutrient solution with different pH values (4.5, 5.0, 5.5, 6.0, 6.5, 7.0, 7.5, and 8.0). After 5 days, a 10 ml suspension of *R. solanacearum* (OD_600_ ≈ 1.0) cultured overnight was poured into each flask. Three days after inoculation, root and leaf samples were collected (0.1 g). Then, the total sample RNA was extracted and reverse-transcribed into cDNA. The primers used in this study are listed in Table 1. Real-time RT-PCR analyses were performed to evaluate the expression profile of the genes *E3ligase, NtAcc oxidase, NtPR 1a/c*, and *Thaumatin* using CXF96 Manager (Bio-Rad). *UBI3* was stably expressed in different conditions and was used as a housekeeping gene (Zhao D. et al., 2009). The total reaction volume of 20 μl included 1 μl of cDNA, 10 μl of Sso Fast™ EvaGreen® Supermix (Bio-Rad), 1 μl of each primer, and 7 μl of RNase-free water. The RT-PCR program was: 95°C for 3 min followed by 40 cycles of 95°C for 10 s and 60°C for 20 s. The relative expression was calculated using the ΔΔCq method.

### Application of wood ash and lime

Field experiments using wood ash and lime to improve the soil pH were conducted in 2012 and 2014 in Qianjiang (2012) and Pengshui (2014), Chongqing, China. One day before transplanting tobacco seedlings (Yunyan97) from floating polystyrene trays to the field, the soil was pretreated with 750 or 1,500 kg/ha of wood ash and lime. The wood ash was a 1:1 mixture of charcoal ash and corn straw ash. Lime was purchased from a local factory. Each treatment plot was randomly designed with an area of ~60 m^2^ (length × width = 12 × 5), and 100 tobacco plants were distributed in each plot. Each treatment was replicated three times. Chemical fertilizers were used at the same level for all treatments. Prior to treatment, the surface zone soil pH (0–15 cm depth) was measured using a soil pH temperature meter (HI199121, Hanawode, Beijing). Soil pH was recorded every fifth day (in 2012) or sixth day (in 2014) after wood ash and lime treatment using the same method. The disease index was recorded every fifth day based on a 0 to 9 scale, as previously described, once bacterial wilt symptoms were observed in the control treatment. The control efficiency (CE) was calculated as CE = ([Disease index in control − disease index in treatment]/disease index in control) × 100%, as previously reported (Guo et al., 2004).

### Data analysis

The data were analyzed using Microsoft Excel 2007 and SPSS 17.0. Statistical significance was determined using one-way analysis of variance (ANOVA), Duncan's multiple range test (DMRT) and independent-samples *t*-test; a *p*-value < 0.05 was considered significant.

## Results

### Significant soil acidification in bacterial wilt fields

**Figure 1:**
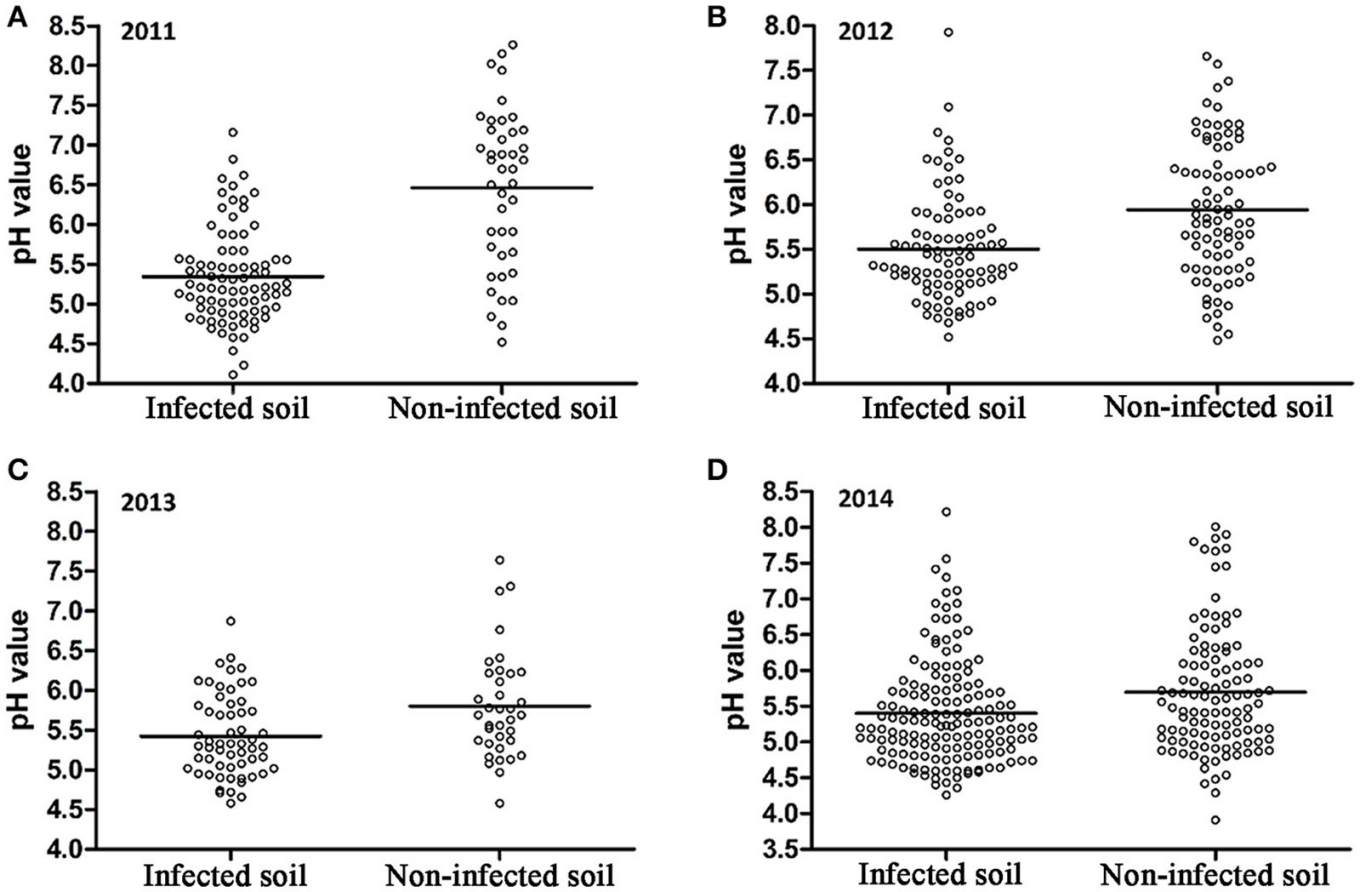
**pH distribution of bacterial wilt infected soil and non-infected soil from 2011 to 2014**. A total of 84 infected soil samples and 42 non-infected soil samples were collected in 2011 **(A)**; 89 infected soil samples and 83 non-infected soil samples were collected in 2012 **(B)**; 58 infected soil samples and 36 non-infected soil samples were collected in 2013 **(C)**; 151 infected soil samples and 109 non-infected soil samples were collected in 2014 **(D)** in southern China. Solid lines indicate the average pH of each group.

**Figure 2:**
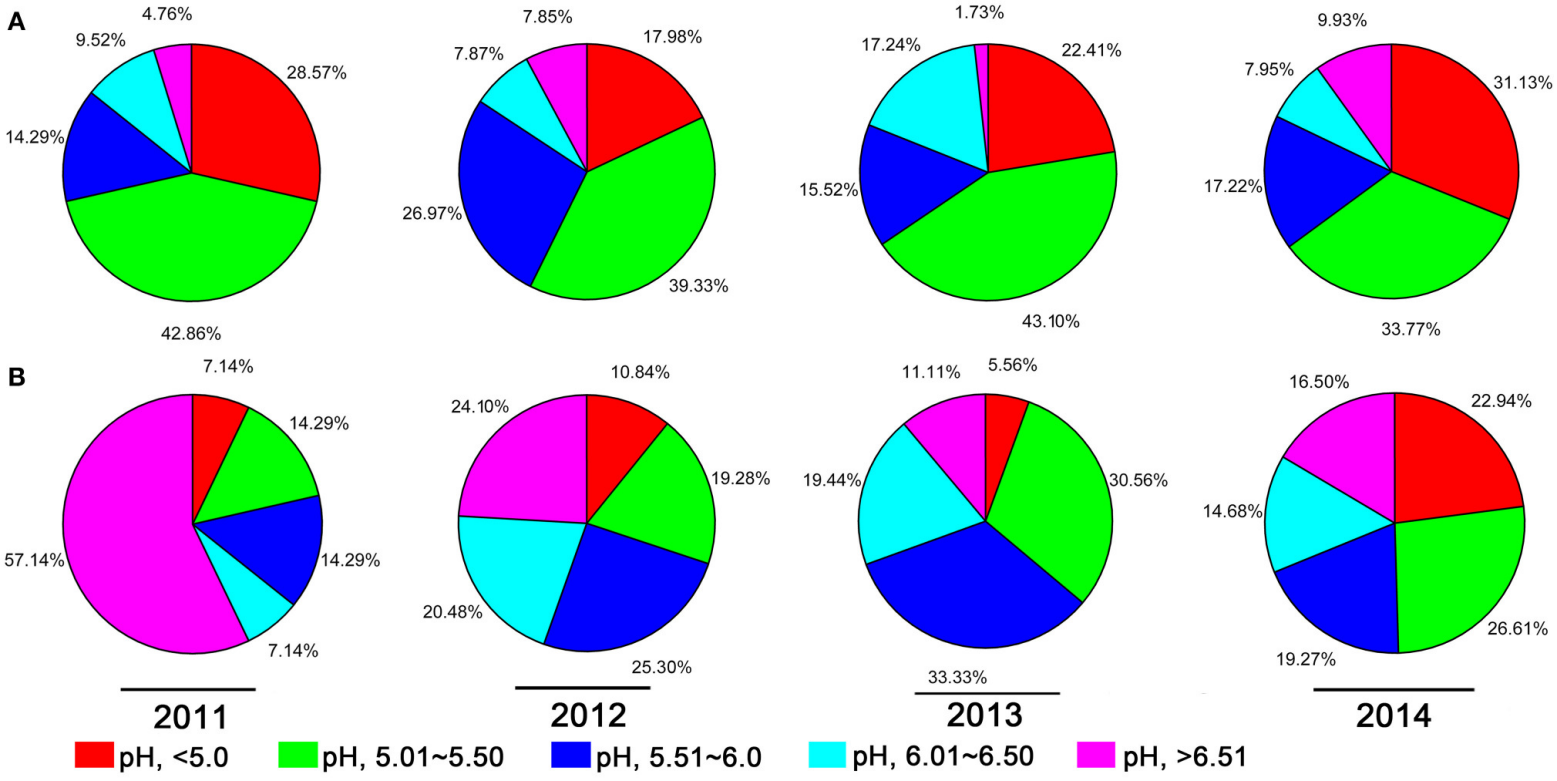
**The proportion of different pH intervals in bacterial wilt infected soil (A)** and non-infected soil **(B)** from 2011 to 2014. Five pH intervals (pH equal to or less than 5.0, pH from 5.01 to 5.50, pH from 5.51 to 6.00, pH from 6.01 to 6.50, and pH equal to or greater than 6.51) were used; pH intervals are indicated by color.

A 10-year continuous tracking survey conducted by our laboratory members showed that soil-borne plant disease bacterial wilt was becoming more serious every year (data not shown). To determine whether the aggravation of bacterial wilt in recent years is associated with significant soil acidification, large-scale sampling was performed from 2011 to 2014, and the pH distributions of infected soils and non-infected soils were compared (Figure 1). The pH in infected soils had a wide distribution, ranging from 4.08 (Figure 1A) to 8.22 (Figure 1D). In non-infected soils, the lowest pH was 3.91 (Figure 1D) and the highest was 8.26 (Figure 1A). These results suggest that both the host and the pathogen have a wide pH range for normal survival and growth. The results based on an overall comparison of 382 infected soils and 270 non-infected soils collected during the 4-year investigation showed that the average pH value of infected soils (pH = 5.42) was much lower than that of non-infected soils (pH = 5.90). Additionally, the average pH of infected soils in each year was less than 5.50. We analyzed the proportion of different pH ranges in infected soils and non-infected soils (Figure 2). In infected soils, the proportion of soil pH values less than 5.50 was 71.43, 57.31, 65.51, and 64.90% in 2011, 2012, 2013, and 2014, respectively. In contrast, a much smaller proportion of soils (21.43, 30.12, 36.12, and 49.55% in 2011, 2012, 2013, and 2014, respectively) had a pH less than 5.50 in non-infected soils. These results indicate significant soil acidification in bacterial wilt fields.

### Soil pH is an important environmental factor for bacterial wilt development in fields

**Figure 3:**
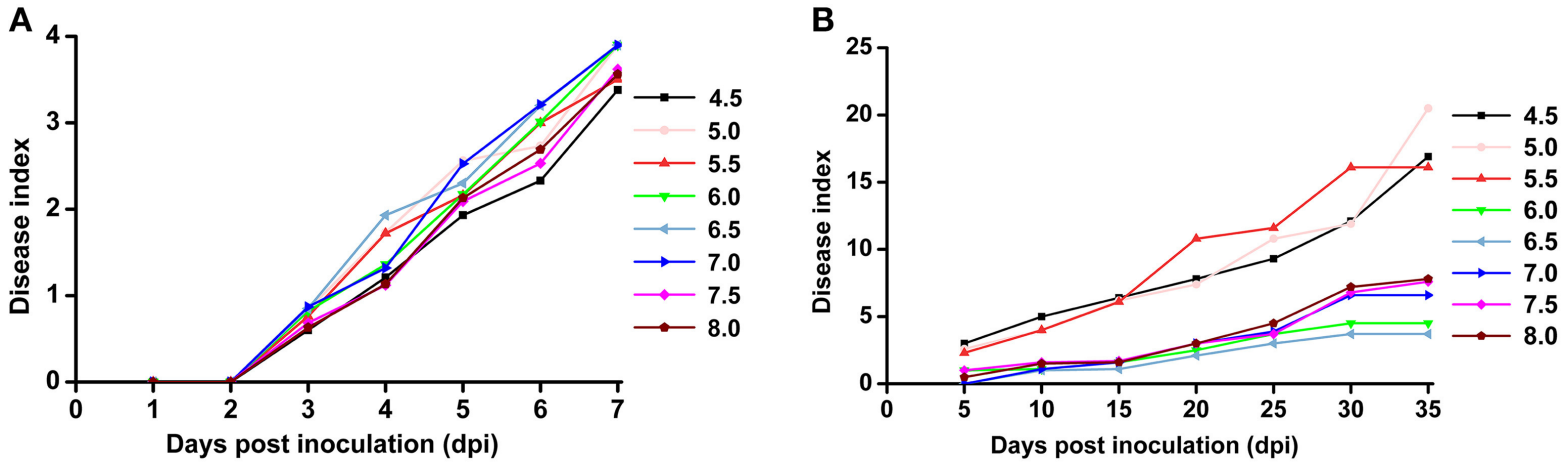
**Bacterial wilt disease development under different pH conditions**. **(A)** Indoor pot experiment. Six-week-old tobacco seedlings (Yunyan 97) grown in autoclaved soil medium with different pH were inoculated with wild-type *R. solanacearum* strain CCT011 by soil drenching. Disease progression was recorded daily using a 0 to 4 disease index scale (0, no symptoms; 1, 1 to 25% of leaves wilted; 2, 26 to 50% of leaves wilted; 3, 51 to 75% of leaves wilted; 4, 76% to 100% of leaves wilted). Each point represents the average disease index of 15 plants. **(B)** Field pot experiment. Tobacco plants (Yunyan 97) grown in natural field soils with different pH were inoculated with *R. solanacearum* CCT011 by soil drenching. Disease symptoms were recorded every fifth day using a disease index scale of 0 to 9 (0, no wilt symptoms; 1, less than half of the leaves on disease side wilted; 3, half to two-thirds of the leaves on disease side wilted; 5, more than two-thirds of the leaves on disease side wilted; 7, all the leaves on disease side wilted; 9, the whole plant died). Each point represents the average disease index of 15 plants.

Based on the previous observation that the average soil pH in bacterial wilt fields was significantly lower, we were interested in the direct effect of soil pH on bacterial wilt disease progression. We performed an inoculation assay under different pH conditions (4.5–8.0 with an interval of 0.5) in climate-controlled room. Briefly, plants were grown in autoclaved seedling soil medium that did not contain any microbial communities and were watered with pH-adjusted water. An *R. solanacearum*-type strain originally isolated from a field with soil pH of 5.93 was used for soil drenching inoculation. No significant difference was observed among different pH conditions according to the 0 to 4 disease index analysis (Figure 3A), suggesting that soil medium pH is not important in bacterial wilt development when plants are grown in autoclaved soil without other microbes. Next, we examined disease development under different pH conditions in the field (Figure 3B). Plants were grown in natural soil containing an established microbial community with artificially adjusted soil pH. Bacterial wilt developed more rapidly and severely when the soil pH was adjusted to 4.5, 5.0, or 5.5 (Figure 3B). Other pH values showed similar delayed disease progression. This suggests that soil pH plays an important role in bacterial wilt disease development under field conditions, and acidic soils (pH 4.5–5.5) promote disease development.

### Acidic conditions favor the growth of *R. solanacearum*

**Figure 4:**
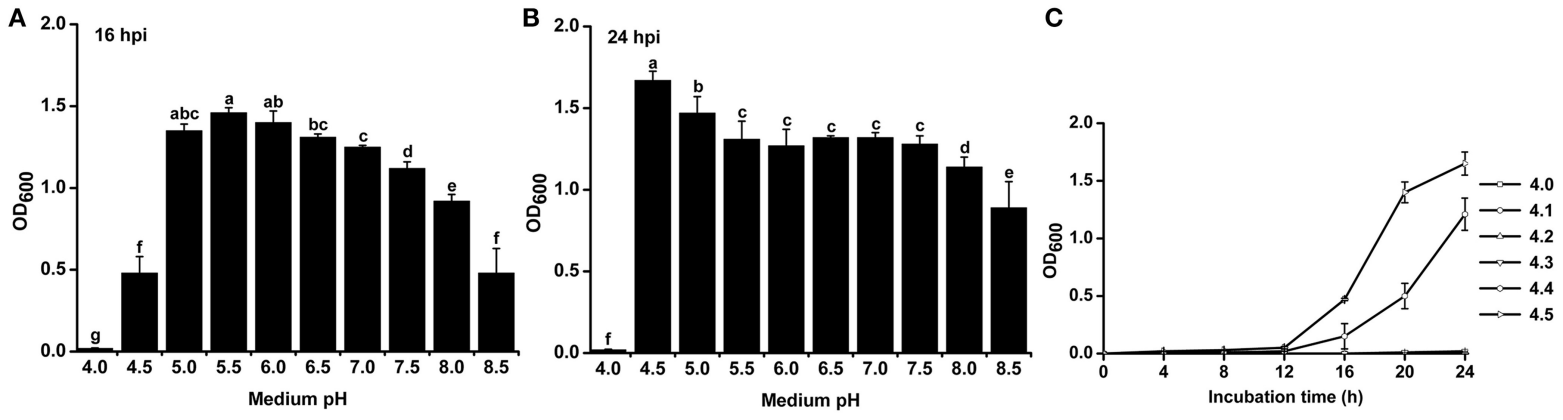
**Growth rate of *R. solanacearum* under different pH conditions**. *R. solanacearum* was grown in B medium with different pH, and the bacterial concentration of each treatment was determined by measuring the OD_600_ value at 16 h post-incubation (hpi) **(A)** and at 24 hpi **(B)**. **(C)** Growth curve of *R. solanacearum* from pH 4.1 to 4.5. Each point represents the average bacterial concentration of three replicates, and error bars indicate the standard deviation. Small letters indicate a significant difference among different pH conditions (ANOVA, Duncan's multiple test).

To study the effect of pH on the growth of *R. solanacearum*, we compared the growth curves in B medium with different pH. The pH was adjusted after autoclaving to avoid pH changes during autoclaving. *R. solanacearum* was able to grow within the pH range 4.5–8.5 (Figure S1). We statistically analyzed the bacterial concentration at 16 h post-incubation (hpi) and 24 hpi. *R. solanacearum* showed the best growth at pH 5.0–6.0 at 16 hpi (Figure 4A). In the following 8 h, *R. solanacearum* grew quickly at pH 4.5. pH 4.5 was the optimal pH for *R. solanacearum* growth at 24 hpi, followed by pH 5.0 (Figure 4B). Alkaline pH conditions (e.g., pH 8.0 and 8.5) significantly slowed the growth of *R. solanacearum*. These results indicate that acidic conditions (pH 4.5–6.5) favors *R. solanacearum* growth.

Since *R. solanacearum* could not grow below pH 4.0 but grew well at pH 4.5, the threshold pH for *R. solanacearum* growth was determined. The growth curve from pH 4.0 to 4.5 with an interval of 0.1 showed that this bacterium could only grow at pH greater than or equal to 4.4 (Figure 4C). Compared to pH 4.5, pH 4.4 inhibited the growth of *R. solanacearum*. This result suggests that pH 4.4 is the minimum threshold for *R. solanacearum* normal growth.

### Influence of different pH conditions on the growth rate and antagonistic activity of antagonistic bacteria

**Figure 5:**
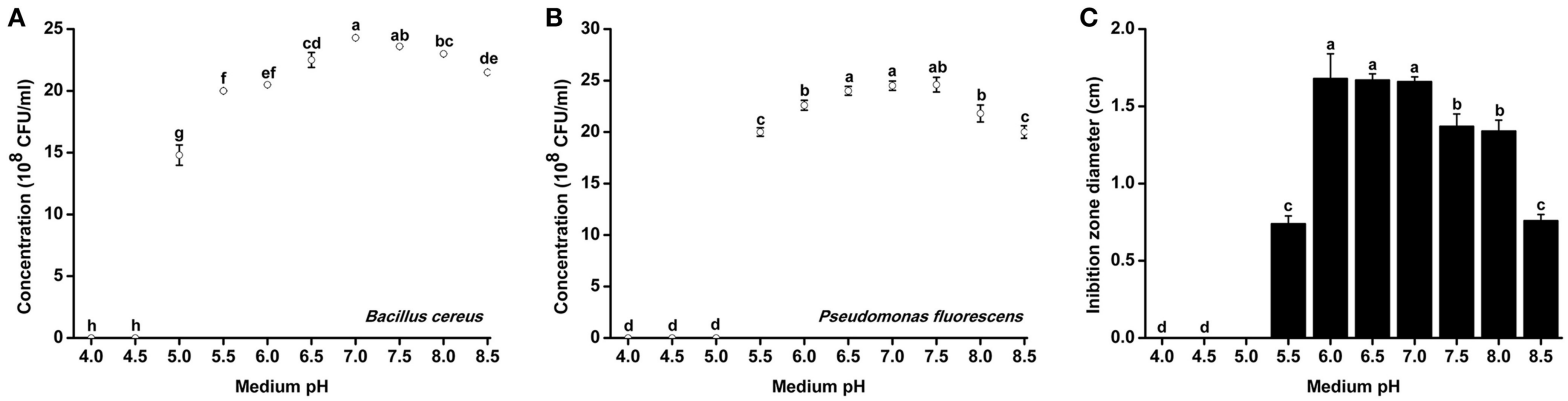
**Growth rate and antagonistic activity of representative antagonistic bacteria under different pH conditions**. *Bacillus cereus* **(A)** and *Pseudomonas fluorescens* **(B)** were grown in Broth medium with different pH for 48 h, and the bacterial concentration was determined using a nephelometer. **(C)** The antagonistic activity of *B. cereus* against *R. Solanacearum* was evaluated using the Oxford cup assay and was determined based on the inhibition zone diameter. Each point represents the average value of three replicates, and error bars indicate the standard deviation. Small letters indicate a significant difference among different pH conditions (ANOVA, Duncan's multiple test).

Since *R. solanacearum* grew much better in acidic conditions than in alkaline conditions, we determined whether pH conditions also affected the growth of antagonistic bacteria. The growth rates of two representative antagonistic bacteria, *B. cereus* and *P. fluorescens*, were measured under different pH conditions. The minimum pH for *B. cereus* normal growth was 5.0, and optimal *B. Cereus* growth occurred at pH 7.0 after 48 h of incubation (Figure 5A). Similar to *B. cereus*, the optimal pH condition for *P. fluorescens* growth was ~7.0. However, *P. fluorescens* could only grow in medium with pH greater than 5.5 (Figure 5B). The growth of both *B. cereus* and *P. fluorescens* was inhibited in acidic conditions (e.g., pH 5.0 and 5.5). These results suggest that neutral and weak alkaline conditions are more conducive for the growth of antagonistic bacteria.

*B. cereus* was further evaluated for its antagonistic activity against *R. solanacearum* under different pH conditions. *B. cereus* exhibited the best antagonistic activity from pH 6.0–7.0 (Figure 5B). The inhibition zone diameter at pH 6.0 reached 1.67 cm, which was considered high antagonistic activity. However, the antagonistic ability of *B. cereus* was significantly weakened when the medium pH was less than 5.5 or greater than 7.5.

### Acidic conditions induced the expression of the virulence genes of *R. solanacearum*

**Figure 6:**
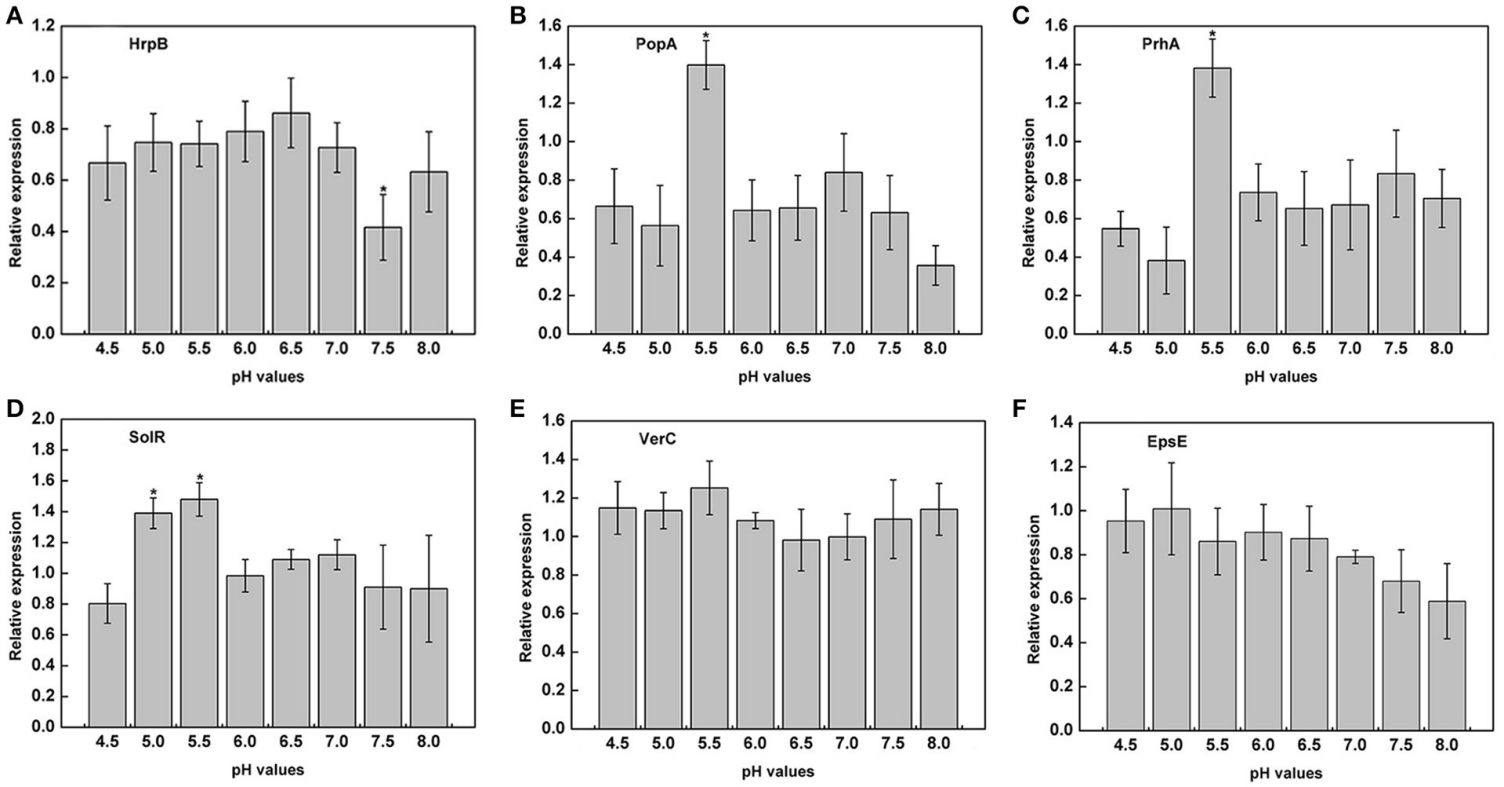
**Expression of virulence genes under different pH conditions**. The relative expression of six virulence genes in B medium with different pH was measured by RT-PCR. **(A)** *HrpB*; **(B)** *PopA*; **(C)** *PrhA*; **(D)** *SolR*; **(E)** *VerC*; **(F)** *EpsE*. *SerC* was used as a control gene to normalize the expression of the target genes. The results are the average value of three independent replicates. Error bars indicate the standard deviation (*P* < 0.05; ANOVA, Duncan's multiple test). The symbol “^*^” above the columns indicate significant differences among the different treatments (Duncan's test, *p* < 0.05).

The previous experiments proved that the growth of *R. solanacearum* could be affected by pH conditions. We measured the expression of virulence genes of *R. solanacearum* grown at pH ranging from 4.5 to 8.0 to determine whether there was a relationship between pH and the activity of virulence genes. The results clearly indicated that the pH of the medium could affect the expression of *PopA, PrhA*, and *SolR*. At pH 5.5, the expression of *PopA, PrhA*, and *SolR* was much higher than that at the other tested pH values (Figures 6B–D). However, extremely acidic or alkaline conditions were not suitable for the expression of *PopA* and *PrhA*. In addition, for *HrpB*, mRNA expression level was lowest at pH 7.5, and the optimum pH ranged from 5.0 to 6.5 (Figure 6A). The tested pH values had no significant effect on the expression of *VerC* and *EpsE* (Figures 6E,F). Nevertheless, the activity of *EpsE* in an acidic environment was higher than that in an alkaline environment, and there was a decreasing tendency along with increasing pH values (Figure 6F).

### Acidic conditions decrease tobacco plant resistance

**Figure 7:**
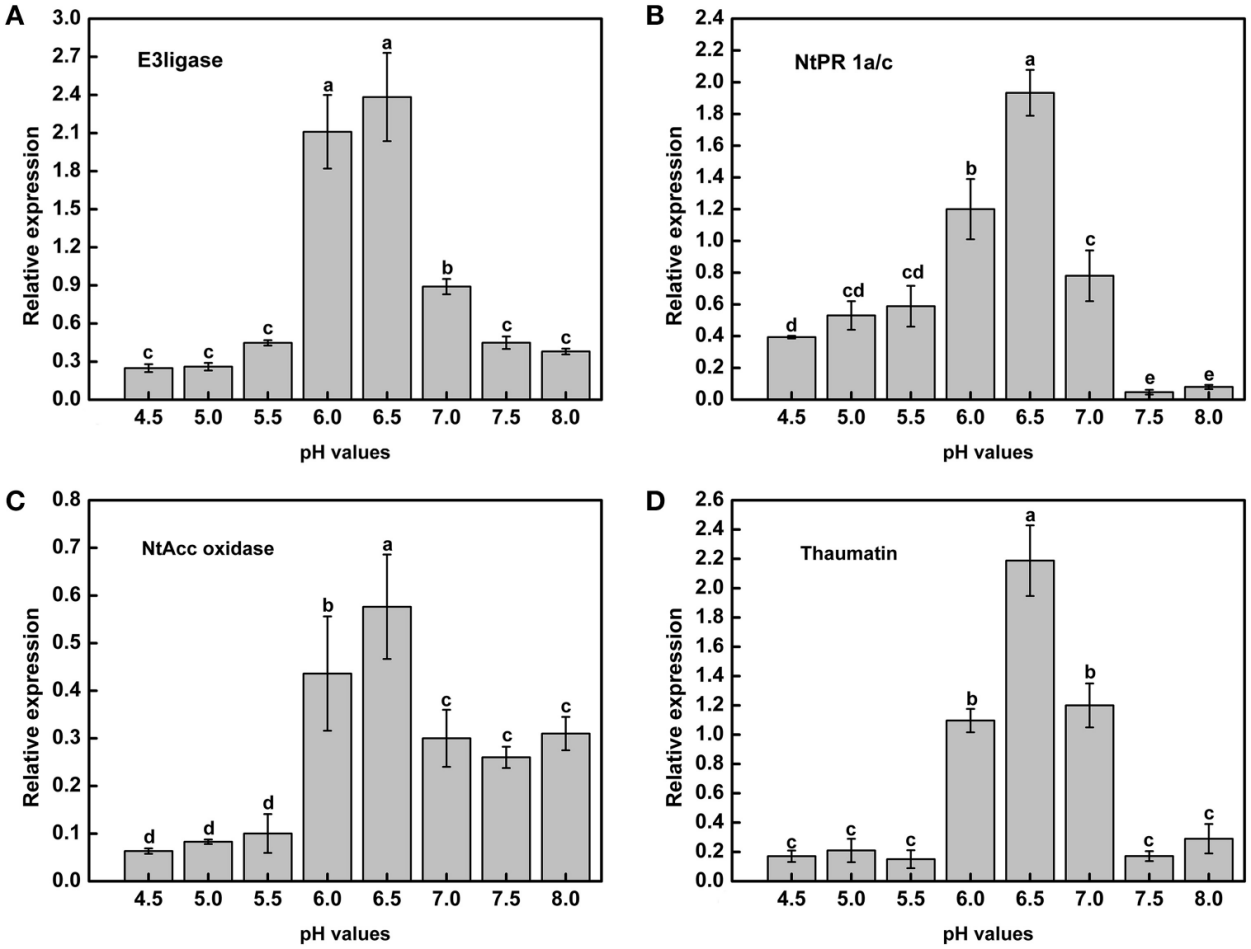
**Expression of tobacco resistance genes induced by *R. Solanacearum* infection under different pH conditions**. **(A)** *E3ligase*; **(B)** *NtPR 1a/c*; **(C)** *NtAcc oxidase*; **(D)** *Thaumatin*. *UBI3* was used as the housekeeping gene to normalize the resistance genes using the ΔΔCq method. The assay was repeated three times. Error bars indicate the standard deviation (*P* < 0.05; ANOVA, Duncan's multiple test). The lowercase letters above the columns indicate significant differences among the different treatments (Duncan's test, *p* < 0.05).

Acidic conditions contributed to the expression of virulence genes in the above experiments. To determine whether tobacco resistance was related to pH, we explored the expression of resistance-related genes under different pH conditions. In addition, the tobacco resistance genes were identified in our laboratory (data not shown). RT-PCR analysis indicated that there was a strong influence of pH on the expression of resistance genes across the gradient (Figure 7). Moreover, mRNA expression of the resistance-related genes was significantly higher in a weakly acidic environment from ~pH 6.0 to 6.5, whereas the expression levels was highest at pH 6.5, with 9.57-, 4.92-, 9.09-, and 12.82-fold up-regulation compared with pH 4.5 (Figures 7A–D). When the pH was less than 6.0 or greater than 7.0, the expression of resistance genes decreased, especially *E3ligase* and *Thaumatin* (Figures 7A,D). In addition, highly acidic conditions significantly inhibited the expression of *NtAcc oxidase* compared with high pH values (pH > 5.5) (Figure 7C). Therefore, extreme soil (pH < 5.5) could significantly reduce the tobacco resistance to *R. solanacearum* from the gene expression difference.

### Application of wood ash and lime improves soil pH and reduces the occurrence of bacterial wilt

**Figure 8:**
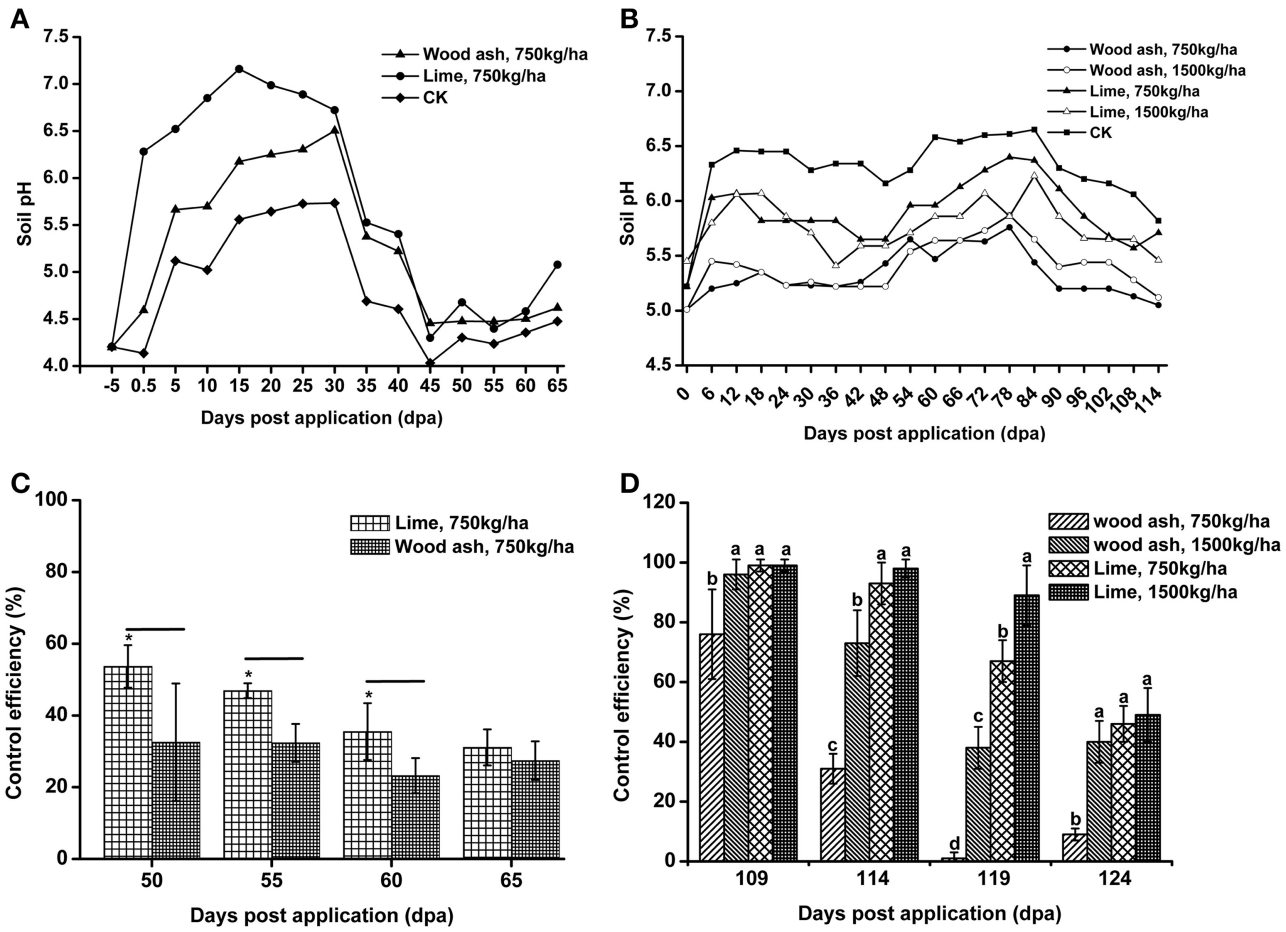
**The application of lime and wood ash improved soil pH and reduced the occurrence of bacterial wilt**. **(A)** Soil pH dynamics after the application of wood ash (750 kg/ha^−1^) and lime (750 kg/ha^−1^) in 2012. The pH value was measured prior to treatment and every fifth day after wood ash and lime treatment. **(B)** Soil pH dynamics after the application of wood ash (750 and 1,500 kg/ha^−1^) and lime (750 and 1,500 kg/ha^−1^) in 2014. The pH value was measured prior to treatment and every sixth day after wood ash and lime treatment. **(C)** The control efficiency (CE) of wood ash and lime toward bacterial wilt in 2012. **(D)** The CE of wood ash and lime toward bacterial wilt in 2014. CE was calculated as CE = ([Disease index in control − disease index in treatment]/disease index in control) × 100%. The CE was the average value of three plots, and small letters indicate a significant difference between different treatments (ANOVA, Duncan's multiple test). The symbols indicate significant differences according to independent-samples *t-test* (^*^p < 0.05).

Lime and wood ash are widely used soil pH remediation agents. These two materials were used to improve the soil pH in acidic bacterial wilt fields. In 2012, wood ash and lime were applied to a very acidic field at a rate of 750 kg/ha. Soil pH was significantly increased 12 days post-application (Figure 8A). After 12 days, soil pH increased in both the remediation agent treated area and control area, followed by a decrease 30 days post-application. The soil pH in the wood ash and lime treated area was much higher than that in the control area throughout the production season. Compared to wood ash, lime resulted in better soil pH improvement.

In 2014, wood ash and lime were used at rates of 1,500 and 750 kg/ha. Soil pH was improved after the application of lime and 1,500 kg/ha of wood ash (Figure 8B). The pH value in the area treated with 1,500 kg/ha of lime was greater than 6.0 within 90 days post-application, while soil pH in the control area fluctuated between 5.0 and 5.5 during the entire production season. We also found that a relatively large amount of lime and wood ash led to better improvement of soil pH. These results suggest that acidic soils could be improved by the application soil pH remediation agents.

To understand the effect of soil pH improvement on disease development, the disease index of the soil pH remediation agent group and control group was recorded, and the CE was calculated. In 2012, both wood ash and lime application reduced the disease index and showed a certain degree of CE (Figure 8C). Lime application, which resulted in better soil pH improvement, exhibited significantly higher CE than wood ash application at 55 and 60 days post-application. The CE of lime and wood ash application, 31.09 and 27.41%, respectively, was very similar at 65 days post-application. In 2014, the application of lime and 1,500 kg/ha of wood ash resulted in good control efficiencies, and the CE of lime application was much better than that of wood ash application (Figure 8D). However, the CE of the 750 kg/ha wood ash application, which almost did not improve soil pH in 2014, was quite low 119 and 124 days post-application. These results suggest that bacterial wilt development is associated with soil pH, and soil pH improvement reduces the occurrence of bacterial wilt.

## Discussion

Recent studies on soil pH changes have revealed significant soil acidification in croplands, grasslands, and alfisols in different parts of China (Guo et al., 2010; Yang et al., 2012). Our data provide evidence that soil acidification occurs in crop fields, especially in mountainous southwest China, where chemical fertilizers have been excessively used in recent decades. Our study also demonstrated that soil acidification is a key environmental factor in the occurrence of bacterial wilt. The results from a 4-year investigation in southern China, where bacterial wilt is severe, showed that the average pH in bacterial wilt fields is much lower than that in healthy fields (Figure 1) and the proportion of areas with pH less than 5.5 in infected soils is much higher than in non-infected soils (Figure 2), suggesting a close correlation between soil pH and the occurrence of bacterial wilt. Although we failed to confirm this correlation in an indoor pot experiment using autoclaved soil medium for plant growth and inoculation assays, the correlation between soil pH and bacterial wilt development was validated by a field pot experiment using natural field soils (Figure 3). The difference in the results occurred because the effect of pH on the pathogen itself was insufficient to cause disease differences in a short period under indoor conditions or because pH does not directly affect bacterial wilt disease development. One possible way that pH affects disease is that soil pH affects the soil microbial activity and thereby affects the progression of bacterial wilt.

*R. solanacearum* is a typical soil-borne plant pathogenic bacterium with a wide host range, from solanaceous crops to *Eucalyptus* spp. (Hayward, 1991). The survival and spread of this pathogen in soil are largely affected by soil chemical properties. The growth curve experiment showed that *R. solanacearum* grew much better in acidic conditions than in alkaline conditions, and the minimum pH for *R. solanacearum* growth was 4.4 (Figure 4), indicating that *R. solanacearum* is well-adapted to an acidic soil environment. This result is inconsistent with a previous study showing that bacterial growth is inhibited at lower pH conditions (Rousk et al., 2009), most likely because *R. solanacearum* is a pathogenic bacterium that is quite different from other soil bacteria. In our field investigation, there were few cases where the soil pH was lower than 4.4 but bacterial wilt still occurred. We speculate that the *R. solanacearum* strains in these fields were under acidic stress for a long time and were thus more tolerant to acidic conditions. Since soil acidification has been a global problem and acidic environments favor *R. solanacearum*, this pathogen may increase its geographical distribution and spread to acidic areas in the near future.

These results raise the question of why soil acidification makes soil susceptible to the outbreak of plant disease, especially soil-borne disease. One hypothesis is that soil acidification affects plant growth and resistance by influencing aluminum accumulation (Rout et al., 2001), the uptake of nutrients (Wang et al., 2000), the activity of soil enzymes (Dick et al., 2000; Graham and Haynes, 2005), and root growth (Haling et al., 2010). In addition, the decrease in soil pH has altered the above-ground plant diversity and productivity in part due to a direct increase in Al^3+^ (Van Den Berg et al., 2011; Chen et al., 2013). When the soil pH decreases to less than 6.0, the soluble Al content increases, which has a deleterious effect on plants, both morphologically and physiologically (Ryan and Delhaize, 2010; Bian et al., 2013). Under acid soils (pH < 5.5) conditions, most essential nutrients cannot be directly taken up by plants, such as Ca, K, Mo, and Mg (Läuchli and Grattan, 2017). Thus, plants will be stressed and less resistant to attack by pathogen in non-optimal pH. In the case of *R. solanacearum*, there is evidence that deficiency of several mineral nutrients will increase severity of tobacco bacterial wilt, such as available K, exchangeable Ca, active Mo (Zheng et al., 2014a). Nevertheless, replenishing tobacco with Mo and Ca nutrition regularly will benefit to enhance the defense ability of tobacco against bacterial wilt (Zheng et al., 2014b). Similarly, the expression of some plant resistance genes are significantly inhibited below pH 5.5 (Figure 7), indicating that soil acidification could lead to a decrease in plant disease resistance. In contrast, *R. solanacearum* could grow much better under weakly acidic conditions, and the expression of the virulence genes of *R. solanacearum* was significantly upregulated at pH values ranging from 5.0 to 5.5 (Figure 6). Based on plant health and *R. solanacearum*, we speculate that soil acidification is an important contributing factor to the outbreak of tobacco bacterial wilt. This conclusion is consistent with previous research that demonstrated that the application of ${\text{NH}}_{4}^{+}$ increases the severity of root disease in acidic soil and decreases the severity of root disease in neutral and alkaline soils (Smiley, 1975).

Soil health has been broadly defined as the capacity of a living soil to function, sustain plant and animal productivity, and promote plant, and animal health (Doran and Zeiss, 2000). Microorganisms are essential components of living soils and are of utmost importance to soil health and can be used as indicators of soil health (Nielsen et al., 2002). Soil pH has a strong positive influence on the soil microbial community structure, richness and evenness index (Rousk et al., 2009). In addition, both the relative abundance and diversity of bacteria in alkaline soil were significantly higher than in acidic soil, nearly doubling between pH 4 and 8 (Rousk et al., 2010a). Our study on two representative antagonistic bacteria, *P. fluorescens* and *B. cereus*, showed that their growth rates and antagonistic activity were affected by pH (Figure 5). Alkaline conditions are beneficial to the growth of antagonistic microorganisms, while growth and antagonistic activity are suppressed under acidic conditions (Figure 5). This result supports the previous finding that bacterial growth is suppressed under low pH conditions (Rousk et al., 2009). Besides, the integration of beneficial microorganisms into agriculture systems regulates the balance of the soil microenvironment, making the microbial community structure more conducive to increased plant and soil health (Avis et al., 2008). Our investigation and field pot experiment indicate that acidic pH environments aggravate the development of bacterial wilt because antagonistic microorganisms are inhibited and the soil microbial balance is broken under acidic conditions, which leads to the loss of beneficial microbes and the ingress of plant pathogens that may have a devastating effect on plant health. Comparison of the microbial community composition in acidic infected soils and healthy soils using 16S rRNA marker gene sequences (Langille et al., 2013) will reveal the precise mechanism of how soil acidification affects the soil microbial community and soil-borne plant diseases.

The use of soil pH amendments is a potent way to improve soil pH. Our data show that soil pH is increased after the application of wood ash (Figure 8). Wood ash has been reported as an efficient soil pH remediation agent (Brunner et al., 2004; Shi et al., 2015). It can decrease soil acidification and affect the microbial properties and plant growth in acidic soils (Zimmermann and Frey, 2002). We found that the effect of wood ash on improving soil pH depends on the application rate and the original soil pH. Compared to wood ash, the application of lime had a stronger effect on increasing soil pH. Lime has also been shown to decrease soil acidification in forest soils and to affect the root growth of barley seedlings growing in acidic soil (Haling et al., 2010; Hu et al., 2016). Based on the pH adjustment ability, lime has great potential for soil acidification repair. We found that the bacterial wilt disease index decreased when the soil pH was improved using lime and wood ash. Studies have shown that the application of lime or alkaline fertilizer provides good CE toward bacterial wilt (Zheng et al., 2013). Our data support the finding of previous studies that lime is a good soil remediation agent and confirm that bacterial wilt is more serious in acidic soils.

In summary, our work demonstrates that soil acidification aggravates the occurrence of bacterial wilt and soil pH improvement with lime and wood ash reduces the disease index of bacterial wilt. Based on our further experiments, the most likely explanation is that the pathogen responsible for bacterial wilt is well-adapted to acidic conditions, while the growth and antagonistic activity of antagonistic microbes are suppressed in lower pH conditions. In general, this study reminds us to pay more attention to the soil acid and alkali balance in controlling bacterial wilt, particularly when soil pH is lower than 5.5.

## Author contributions

SL conceived and designed the experiments, performed the analysis, and wrote the manuscript. JW, YL, LY, SZ, and WD contributed to collect the soil samples and analyzed the data. CX and WD designed the experiments and modified the manuscript.

### Conflict of interest statement

The authors declare that the research was conducted in the absence of any commercial or financial relationships that could be construed as a potential conflict of interest.

## Supplementary material

Figure S1 **Growth curve of *R. solanacearum* under different pH conditions**. The OD of each treatment is the average of three replicates. The error bars indicate the standard error of the mean from three replicates.
