# Supplementary material for: Soil Acidification Aggravates the Occurrence of Bacterial Wilt in South China
Source: Front Microbiol. 2017 Apr 25;8:703. doi: 10.3389/fmicb.2017.00703 (PMC5403937; doi:10.3389/fmicb.2017.00703)
Supplement: Figure S1 — Growth curve of R. solanacearum under different pH conditions. The OD of each treatment is the average of three replicates. The error bars indicate the standard error of the mean from three replicates. [file DataSheet1.docx]

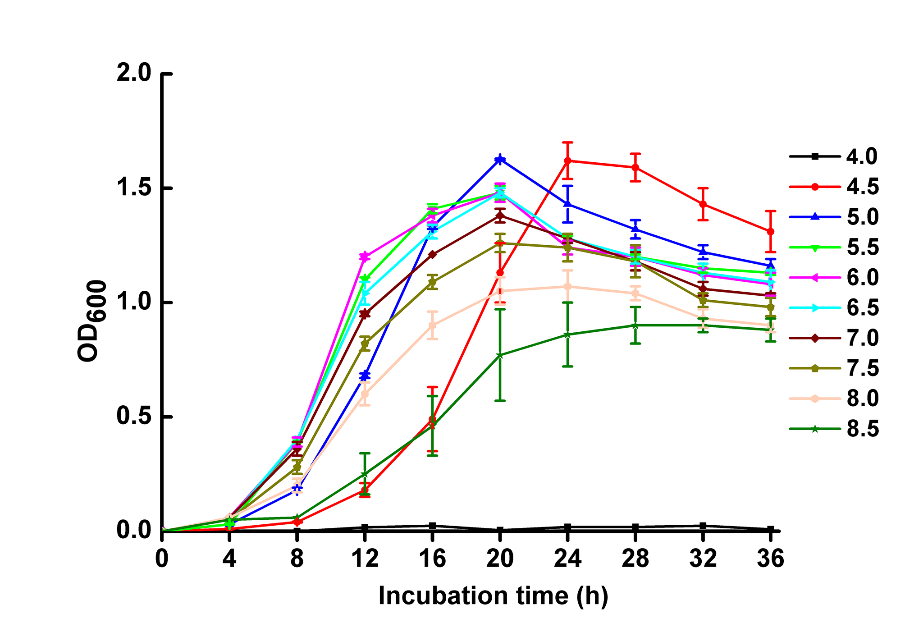


Fig. S1. Growth curve of *R. solanacearum* under different pH conditions. The OD of each treatment is the average of three replicates. The error bars indicate the standard error of the mean from three replicates.
